# Bilateral Linear Porokeratosis Treated With Topical Cholesterol 2%/Lovastatin 2%

**DOI:** 10.7759/cureus.27540

**Published:** 2022-07-31

**Authors:** Darlene Diep, Tyler Janitz, Kamilah S Kannan, Alison Crane, Bineetha Aluri, Kevin Wright, William Baker

**Affiliations:** 1 Medicine, Burrell College of Osteopathic Medicine, Las Cruces, USA; 2 Biological Sciences, University of Georgia, Athens, USA; 3 Dermatology, US Navy, San Diego, USA

**Keywords:** lovastatin, blashko, mevalonate, cholesterol, bilateral linear porokeratosis

## Abstract

Linear porokeratosis is a cutaneous disorder that typically presents in a unilateral linear formation. While the exact cause of linear porokeratosis is unknown, it is thought to be a downstream effect of disrupted cholesterol synthesis and mevalonate accumulation.

Our patient is a 61-year-old male with an unusual case presentation of bilateral linear porokeratosis. He had failed numerous standard therapies. Pathologic examination of a skin biopsy was consistent with bilateral linear porokeratosis. Through a PubMed search, there have been limited reported cases of unilateral linear porokeratosis, but there have not been any reported cases of bilateral linear porokeratosis.

There are currently limited therapies with satisfactory outcomes for variants of porokeratosis. While there are some studies on the topical application of cholesterol/lovastatin, limited studies have been performed on the linear form. Our study evaluates the efficacy of compounded topical cholesterol 2%/lovastatin 2% ointment on bilateral linear porokeratosis. The patient demonstrated a significant reduction of porokeratotic lesions on the treated arm compared to the untreated arm. Cholesterol/lovastatin is alternative therapy that can be considered in the treatment of linear porokeratosis and other porokeratosis variants.

## Introduction

Linear porokeratosis is a skin condition in which abnormal keratinized cutaneous lesions are arranged in a linear formation, usually following dermatomes or Blaschko lines. Clinically, these lesions appear as red patches or plaques and are typically unilateral in nature [1]. Histologically, porokeratoses are characterized by the cornoid lamellae, which present as vertical columns of parakeratosis and dyskeratotic cells found in the granular layer of the epidermis [2]. Porokeratosis leads to an increased risk of keratinocyte malignant transformation that can lead to skin cancer, with the most common being squamous cell carcinoma [3]. Current therapies have variable or unsatisfactory outcomes. We present an unusual case of a bilateral variant of linear porokeratosis.

## Case presentation

A 61-year-old male presented to his family medicine physician with asymptomatic erythematous plaques on the bilateral upper extremities of over 50 years duration. The condition was first realized as a quarter-sized hyperkeratotic patch on the right proximal upper extremity at the age of three. As he grew older, the lesion began to spread across his right upper extremity and proceeded to involve his left upper extremity. The results of his first skin biopsy at age nine were inconclusive.

**Figure 1:**
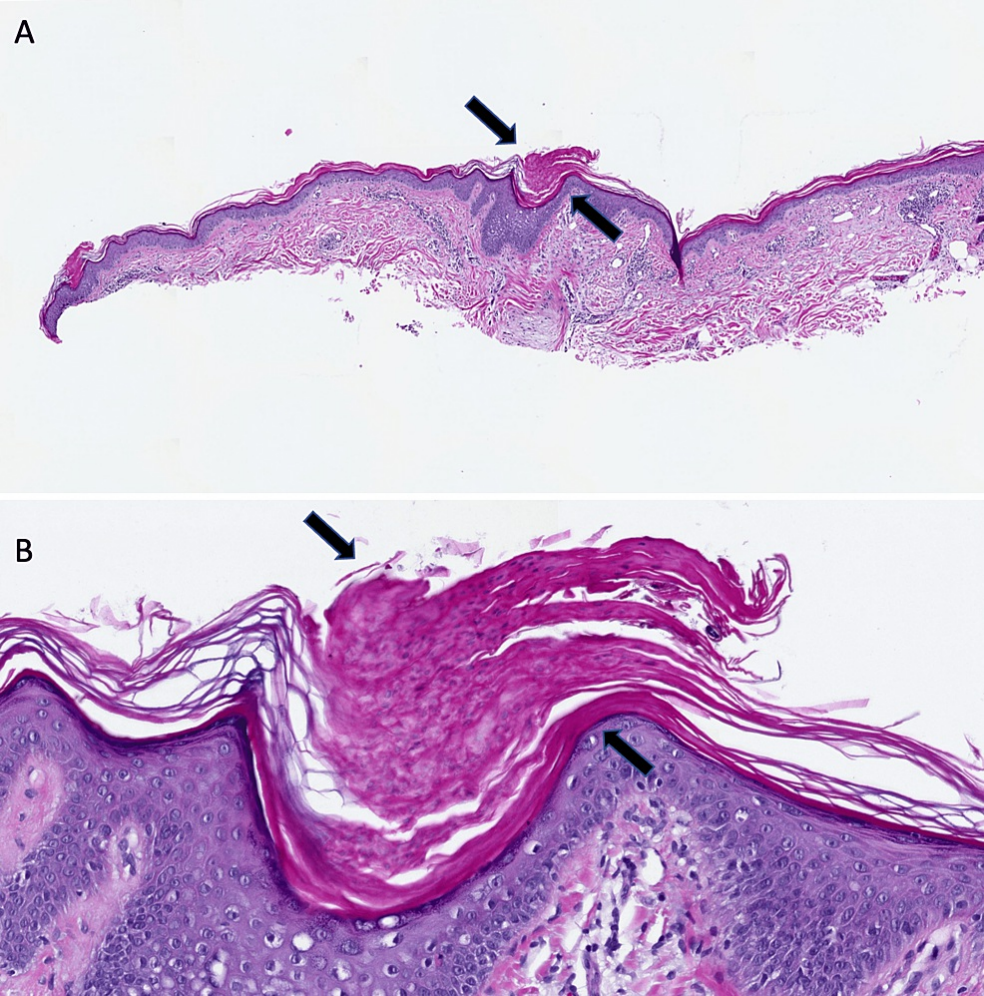
Microscopic presentation of linear porokeratosis Lower (A) and higher (B) power views of the biopsy specimen showing cornoid lamellae (black arrows), parakeratosis, and dyskeratosis in the epidermis (hematoxylin-eosin: A=x 1 and B=x10).

At age 55, the patient was initially diagnosed with dermatitis and was given clobetasol 0.05% ointment but was unable to tolerate treatment due to the burning sensation. He was then prescribed calcipotriene 0.005% cream and diclofenac 1% gel for six months, which had no effect. The patient was subsequently sent to a dermatologist, who performed a skin biopsy. The clinical features and histopathologic findings of cornoid lamellae were consistent with bilateral linear porokeratosis (Figure 1).

**Figure 2:**
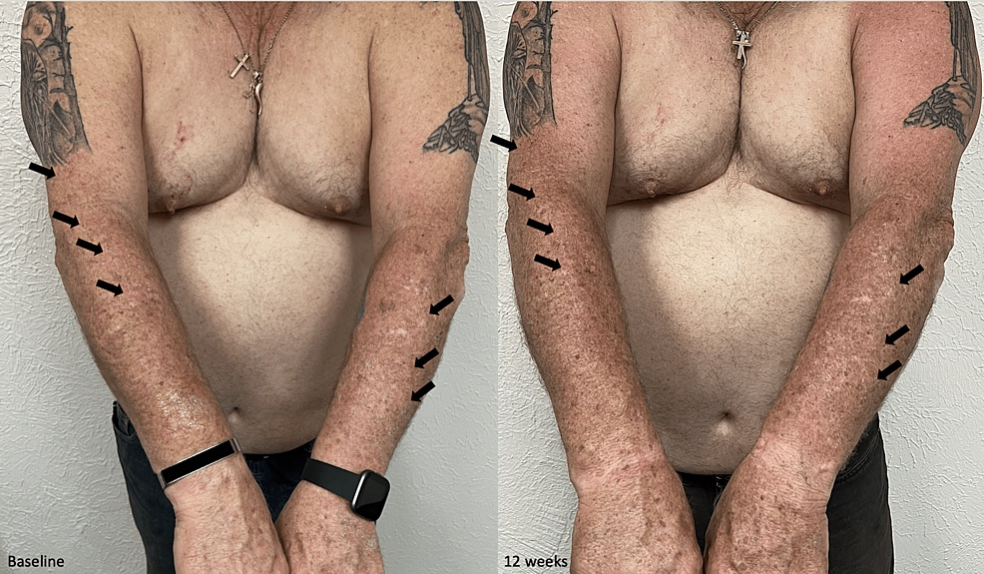
Bilateral linear porokeratosis treated with topical cholesterol/lovastatin ointment Before treatment, the patient presented with bilateral erythematous and hyperkeratotic plaques distributed in a linear form in the upper extremities. After 12 weeks of treatment of the right arm with topical cholesterol/lovastatin, the patient achieved a smoother texture with some residual post-inflammatory hyperpigmentation compared to baseline. The untreated left arm remained unchanged.

After obtaining informed consent, we initiated cholesterol 2%/lovastatin 2% ointment therapy. The patient was instructed to topically apply the treatment to his right upper extremity twice daily for 12 weeks. The left arm was untreated and used as the control. The patient was examined at four-week intervals for up to three months to assess the clinical response. At the end of the twelve-week trial, there was a significant reduction in erythema and scaling compared to the baseline and the untreated arm (Figure 2).

**Figure 3:**
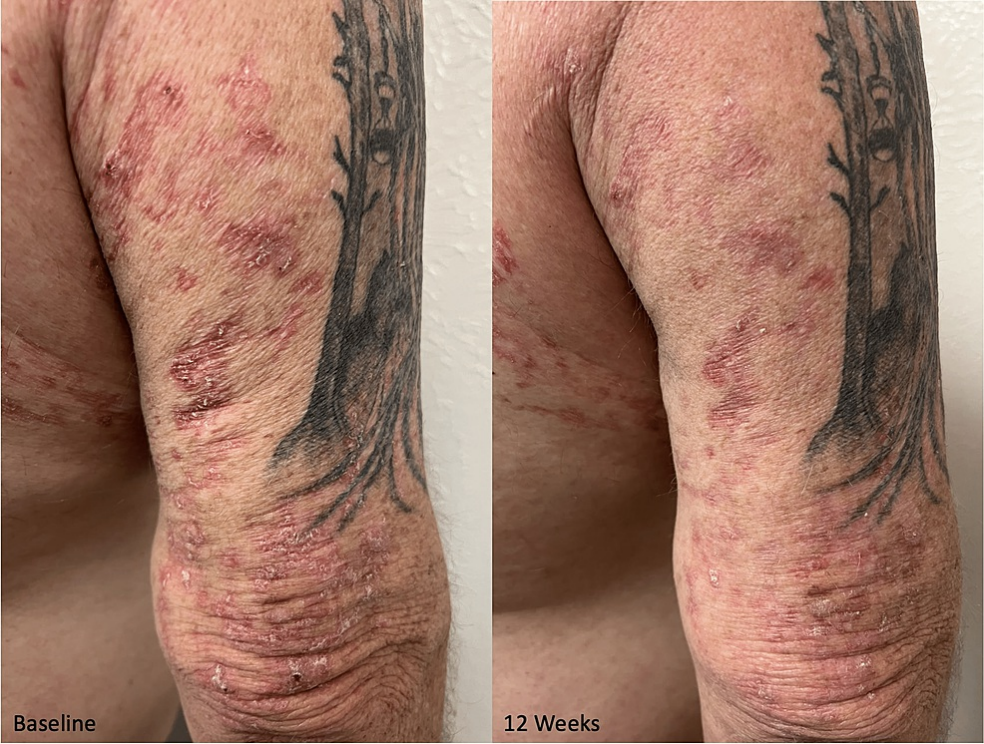
Closer views of linear porokeratosis before and after treatment with cholesterol/lovastatin ointment Visible improvement of the hyperkeratotic plaques on the posterior right arm after 12 weeks of treatment with cholesterol/lovastatin ointment compared to baseline.

Some hyperkeratotic lesions persisted but were smaller in size (Figure 3). The remaining erythema was attributed to residual inflammation from the primary process; it is expected to continue to resolve on its own and fade in color. The lesions on the left arm were unchanged. Throughout the trial, the patient denied any adverse effects of the medication, including pain, itching, and swelling.

## Discussion

Cholesterol/lovastatin was selected as pathway-directed therapy. Early studies suggest therapy targeting the pathogenesis had marked success at improving linear porokeratosis, as observed by the reduction of plaques and erythema in our patient. Atzmony et al. demonstrated two cases of linear porokeratosis, along with other variants of porokeratosis, which had improved plaques and erythema after five and 12 weeks of treatment [4]. Blue et al. successfully treated a case of linear porokeratosis in a pediatric patient, which nearly resolved the lesions and erythema [5]. 

A current theory proposes a two-hit, loss-of-function mutation in the phosphomevalonate kinase (PVMK) and mevalonate diphosphate decarboxylase (MVD) genes of the cholesterol synthesis pathway [6]. These mutated genes inhibit cholesterol synthesis. Since cholesterol contributes to the integrity of the stratum corneum, cholesterol depletion causes premature apoptosis of the keratinocytes, leading to the formation of porokeratosis.

Cholesterol monotherapy has been shown to be ineffective [4]. PMVK and MVD mutations prevent the breakdown of mevalonate within the cholesterol pathway, leading to an accumulation of toxic metabolites. These metabolites have been shown to initiate the innate immune response and increase cytokine production, which can contribute to the development of porokeratosis [7]. Statins prevent mevalonate accumulation by inhibiting 3-hydroxy-3-methylglutaryl-CoA reductase (HMG-CoA reductase) and thus attenuate the mevalonate toxic metabolite accumulation and proinflammatory response. 

The improvement of the appearance of linear porokeratosis treated with cholesterol/lovastatin supports the theory of the disrupted cholesterol synthesis pathway. While the cholesterol is replenished in the skin barrier, lovastatin prevents mevalonate accumulation to downregulate the inflammation that is thought to cause the porokeratosis lesions.

## Conclusions

There are limited therapeutic options for linear porokeratosis. A man presented with a rash of the bilateral upper extremities that was refractory to treatment with topical steroids, calcipotriene, and diclofenac. Consideration of the clinical presentation and histopathological evaluation revealed the diagnosis of linear porokeratosis. The topical application of cholesterol/lovastatin to the more affected arm demonstrated efficacy in reducing the plaques and appearance of the lesions at 12 weeks compared to the baseline. 

Cholesterol and lovastatin offer a relatively inexpensive alternative treatment option that may have better outcomes than current standard therapies. The noticeable improvement and tolerability suggest that topical cholesterol 2%/lovastatin 2% ointment should be considered in the treatment of linear porokeratosis.
